# The residual cancer burden index as a valid prognostic indicator in breast cancer after neoadjuvant chemotherapy

**DOI:** 10.1186/s12885-023-11719-z

**Published:** 2024-01-02

**Authors:** Xin Xu, Wei Zhao, Cuicui Liu, Yongsheng Gao, Dawei Chen, Meng Wu, Chao Li, Xinzhao Wang, Xiang Song, Jinming Yu, Zhaoyun Liu, Zhiyong Yu

**Affiliations:** 1 Tianjin Medical University Cancer Institute & Hospital,National Clinical Research Center for Cancer, Tianjin’s Clinical Research Center for Cancer, Key Laboratory of Breast Cancer Prevention and Therapy, Tianjin Medical University, Ministry of Education, Key Laboratory of Cancer Prevention and Therapy, Tianjin, 300000 China; 2 Affiliated Hospital of Jining Medical University, Jining, 272060 China; 3 Liaocheng People’s Hospital, Liaocheng, China; 4 Shandong Cancer Hospital and Institute, Shandong First Medical University and Shandong Academy of Medical Sciences, Jinan, Shandong 250117 People’s Republic of China; 5 Departments of Oncology, The Second Affiliated Hospital of Shandong First Medical University, Shandong Province, Tai’an, 271000 China

**Keywords:** Residual cancer burden, Neoadjuvant chemotherapy, Breast cancer, Pathologic complete response, Miller-Payne grading

## Abstract

**Purpose:**

The residual cancer burden index (RCB) was proposed as a response evaluation criterion in breast cancer patients treated with Neoadjuvant Chemotherapy (NAC). This study evaluated the relevance of RCB with replase-free survival (RFS).

**Methods:**

The clinical data of 254 breast cancer patients who received NAC between 2016 and 2020 were retrospectively collected. The relationship between clinicopathologic factors and RFS was evaluated using Cox proportional hazards regression models. RFS estimates were determined by Kaplan–Meier(K-M) analysis and compared using the log-rank test. Multivariate logistic regression analysis was used to evaluate the risk factors associated with RCB. Receiver operating characteristic (ROC) curves showed the potential of the RCB and MP grading systems as biomarkers for RFS.

**Results:**

At a median follow-up of 52 months, 59 patients(23.23%) developed relapse. Multivariate Cox regression showed that older age (*P* = 0.022), high Pathologic T stage after NAC (*P* = 0.023) and a high RCB score(*P* = 0.003) were risk factors for relapse. The outcomes of the multivariate logistic analysis indicated that RCB 0 (pathologic complete response [pCR]) was associated with HER2-positive patients (*P* = 0.002) and triple-negative breast cancer (TNBC) patients (*P* = 0.013). In addition, the RCB and MP scoring systems served as prognostic markers for patients who received NAC, and their area under curves (AUCs) were 0.691 and 0.342, respectively.

**Conclusion:**

These data suggest that RCB can be equally applied to predict RFS in Chinese patients with NAC. The application of RCB may help guide the selection of treatment strategies.

**Supplementary Information:**

The online version contains supplementary material available at 10.1186/s12885-023-11719-z.

## Introduction

The prevalence of breast cancer has continuously increased worldwide over the past few decades, with a particularly drastic increase in developing countries [1, 2].Breast cancer has emerged as the most prevalent female malignant neoplasia, with morbidity predicted to continue to increase in the future [3]. GLOBOCAN 2020 latest cancer burden data show breast Cancer has replaced lung cancer as the most common cancer in the world [4]. All the number of new cases of global breast cancer reached 2.26 million, accounting for the fifth most common cause of cancer death overall. There were 416 000 new breast cancer cases and deaths in China 117,000 cases, ranking the first highest incidence of female cancer [5]. Although advancements have been made in early detection and aggressive treatment in recent decades, the health quality and prognosis of breast cancer patients remain poor.

Currently, Neoadjuvant chemotherapy (NAC) is recognized as an indispensable treatment option for locally advanced breast cancer [6, 7].The principal role of NAC is to downstage cancers, so that inoperable patients can undergo surgery, or patients who are not suitable for breast-conserving surgery (BCS) can obtain breast-conserving opportunities to satisfy their aesthetic needs. In addition, the response to NAC can be used to evaluate drug sensitivity and guide follow-up adjuvant therapy [8–10]. Patients who achieve pathologic complete response (pCR) after NAC may have a long-term survival benefit, although differences have been observed among breast cancer subtypes [11]. Many breast cancer patients who cannot reach pCR after NAC, and the prognosis of non-pCR patients greatly varies [12, 13].Therefore, to maximize the effect of neoadjuvant therapy regimens in distinct breast cancer subtypes, a more precise pathological evaluation system is urgently required to guide clinicians to develop personalized treatment protocols and improve the prognosis of patients.

The residual cancer burden (RCB) index is a scoring system for assessing residual lesions after NAC based on breast tumors and regional lymph nodes proposed in 2007 [14]. Subsequently, much clinical evidence from America and Europe has proven that the RCB system is effective, repeatable and useful for the pathological evaluation of different subtypes of breast cancer after treatment [15–18]. The RCB system is considered as a long-term prognostic indicator for NAC treatment and has been demonstrated to be a better predictor of overall survival than most evaluation systems [19]. RCB index and classification could help determine the most appropriate treatment plans for patients with all breast cancer subtypes. Residual cancer burden (RCB) continuous index and classification were independently and strongly prognostic for all breast cancer phenotypes. RCB index also was tightly associated with prognosis over long-term follow-up [20].In recent years, the RCB system has been gradually recognized in Asia, and the 2021 version of the Chinese Society of Clinical Oncology (CSCO) guidelines added the RCB index as a post-NAC evaluation system. Our research mainly aimed to validate the contact between the RCB score and prognosis in the Chinese population by analyzing real-world data.

## Methods

### Patients and data collection

In this retrospective study, we included breast cancer patients who received NAC at Shandong Cancer Hospital and Institute and Liaocheng Peoples Hospital between 2016 and 2020. "Neoadjuvant chemotherapy" was used as the appropriate keyword to search for breast cancer patients via the medical record system. The patients who underwent NAC and were diagnosed with breast cancer for using the patient interface of the hospital electronic medical record system, utilizing the keyword "nejuvant chemotherapy".We excluded patients who did not receive surgery after NAC. Among these patients,223 (87.8%) underwent radical mastectomy, and 31 (12.2%) underwent BCS. All of the patients after BCS had completed radiotherapy. We also gathered clinicopathological data, including the onset age, menopausal status, clinical stage, pretreatment estrogen receptor (ER) and progesterone receptor (PR) levels and human epidermal growth factor receptor 2 (HER2) and Ki-67 statuses, type of operation, posttreatment T stage and N stage, presence of lymphatic vessel invasion (LVI), chemotherapy regimen, targeted therapeutic options, and Miller-Payne grade.

The enrolled patients were classified according to ER,PR and HER2 status as follows: ER-positive or PR-positive and HER2-negative was defined as HR + /HER2-; ER-positive or PR-positive and HER2-positive was defined as HR + /HER2 + ; ER- negative, PR-negative, and HER2-positive was defined as HR-/HER2 + ; ER-negative, PR-negative, and HER2-negative was defined as triple-negative breast cancer (TNBC). ER and PR were positively stained in at least 1% of nuclei. HER2 positivity was defined as an immunohistochemistry score of 3 + or 2 + with HER2 gene amplification by fluorescence in situ hybridization.

### Miller-Payne grading system

The Miller-Payne grading system is routinely used by the two hospitals to assess the pathologic response after NAC. The criteria of classification were as follows.

Grade 1: No change or some alteration to individual malignant cells but no reduction in overall cellularity.

Grade 2: A minor loss of tumor cells(up to 30% loss),but high overall cellularity.

Grade 3: Estimated 30–90% reduction in tumor cells.

Grade 4: A marked disappearance of tumor cells (more than 90% loss of tumor cells) such that only small clusters or widely dispersed individual cells remain.

Grade 5: No malignant cells identifiable in sections from the site of the tumor; only vascular fibroelastotic stroma containing macrophages often remains. However, ductal carcinoma in situ (DCIS) may be present.

### RCB score calculation

Neither of the two hospitals routinely evaluated the pathology by the RCB system. Thus, two pathologists from Shandong Cancer Hospital reevaluated the postoperative pathology of the 254 patients according to the requirements of the RCB evaluation system and input the data into the network calculator (www.mdanderson.org/breastCancer_RCB) to calculate the RCB index. Then, according to the cutoff values of 1.36 and 3.28, the patients were further categorized into four different RCB classes: RCB 0 (equal to pCR), RCB I(minimal burden), RCB II (moderate burden) and RCB III(extensive burden).The specific classification methods were as follows: Pathological complete response (pCR), defined by the exclusion of any residual cancer.RCB score of 0 was defined as pCR, RCB score greater than 0, less than or equal to 1.36 was defined as RCB grade I, RCB score greater than 1.36 and less than or equal to 3.28 was defined as RCB grade II, and RCB score greater than 3.28 was defined as RCB grade III [20].

### Routine survival tracking

The patients were followed up for a long time via outpatient reexamination, telephone and e-mail. All cases received a standard postsurgical records, with scheduled clinical visits and imaging examinations every 3 months during the first year, every 6 months during the subsequent 2 years, and once yearly thereafter.

The primary follow-up endpoint was RFS, with the interval from the operation to the first occurrence of disease relapse and distant metastasis. The Kaplan–Meier survival curve is a commonly used statistical method to assess the probability of survival or occurrence of an event in patients within a specific time frame. In the case of breast cancer patients treated with NAC, the Kaplan–Meier survival curve can be used to evaluate the probability of relapse.

### Statistical analysis

The data analysis was performed with SPSS V.25 and GraphPad Prism 8.0.2.The clinically significant pathological features were screened via a Cox regression model, and the log-rank test was performed. Logistic regression was used to identify the factors associated with pCR. GraphPad Prism 8.0.2 was used to draw the survival curves of RFS and pCR. The diagnostic efficiency was judged by the receiver operating characteristic (ROC) curve, including the area under curve (AUC), specificity and sensitivity. P ≤ 0.05 was defined as statistically significant.

## Results

### Baseline features

**Fig. 1:**
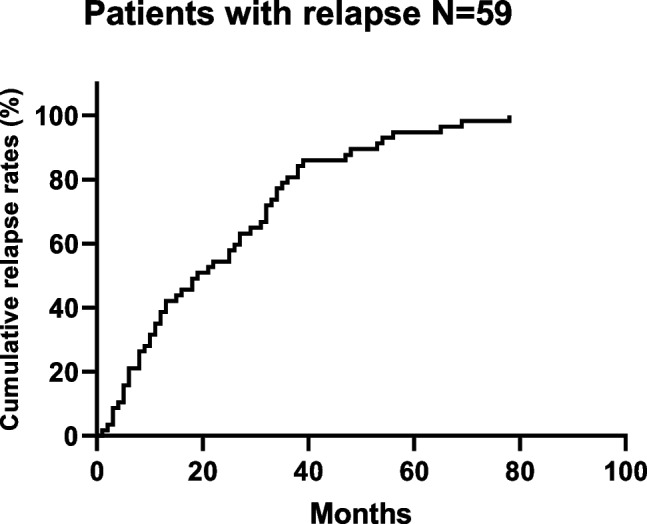
Kaplan–Meier survival curve for relapse in patients treated with NAC

In all, 254 patients with a median follow-up of 52 months were enrolled. A total of 59 patients (23.23%) developed recurrence (Fig. 1 for recurrence survival curve), including local recurrence in 7 patients (2.8%) and distant metastasis in 52 patients (20.5%), 1 patient died of multiple metastases and 1 patient died of brain metastasis.

The median age at the first diagnosis was 49 years (28 to 71 years),with 102 patients (40.2%) being more than 50 years old at the first diagnosis. The clinical stages of all the patients were II or III: 125 patients (49.2%) were stage II, and 129 (50.8%) patients were stage III. The percentage of premenopausal women at first diagnosis was 61.4%. At pretreatment, ER-positive patients accounted for 60.2% (153/254), 116 patients (45.7%) were PR-positive and 99 patients (39.0%) were HER2-positive. All the patients received NAC and were divided into three cohorts: 14 patients (5.5%) were treated with accepted anthracyclines only, 20 patients (7.9%) were treated with accepted taxanes only, and 220 patients (86.6%) were treated with anthracycline and taxane combinations. Among 99 the patients who were HER2-positive, 44 (44.4%) were administered anti-HER2 treatment using trastuzumab, 14 patients (14.1%) selected dual-targeting therapy, and an additional 41 patients (41.4%) rejected receiving any targeted therapies. The RCB scores were judged by two expert pathologists. A total of 59 patients(23.2%) were categorized as RCB 0 (pCR), 33(13.0%) as RCB I, 91(35.8%) as RCB II and 71(28.0%) as RCB III. The patients were divided into four types according to the HR and HER2 status of preoperative biopsy: 107 patients (42.1%) had HR + /HER2- breast cancer, 46 patients (18.1%) had HR + /HER2 + breast cancer,53 patients (20.9%) had HR-/HER2 + breast cancer, and 48 patients (18.9%) had TNBC( as shown in Table 1).

**Table 1:** Baseline characteristics of patients

Patient Characteristics		
Factors	N	Percentage(%)
Overall	254	100.0
RFS event	59	23.23
Age at diagnosis		
≤ 50	152	59.8
> 50	102	40.2
Menopausal status		
premenopausal	156	61.4
postmenopausal	98	38.6
Clinical stage		
II	125	49.2
III	129	50.8
Estrogen receptor		
Negative	101	39.8
Positive	153	60.2
Progesterone receptor		
Negative	138	54.3
Positive	116	45.7
HER2 status		
Negative	155	61.0
Positive	99	39.0
Ki-67 proliferation index		
≤ 20	85	33.5
> 20	169	66.5
Receptor status		
HR + /HER2–	107	42.1
HR + /HER2 +	46	18.1
HR-/HER2 +	53	20.9
TNBC	48	18.9
Type of breast surgery		
Mastectomy	223	87.8
Breast conservation	31	12.2
Pathologic T stage after NAC		
ypT0	122	48.0
ypT1	78	30.7
ypT2	48	18.9
ypT3	2	0.8
ypT4	4	1.6
Pathologic N stage after NAC		
ypN0	118	46.5
ypN1	74	29.1
ypN2	44	17.3
ypN3	18	7.1
lymphatic vessel invasion(LVI)		
Negative	217	85.4
Positive	37	14.6
Neoadjuvant chemotherapy regimen		
Anthracycline	14	5.5
Taxane	20	7.9
Anthracycline + Taxane	220	86.6
Neoadjuvant anti-HER2 therapy regimen		
reject	41	16.1
Single targeted drug	44	17.3
Double targeted drugs	14	5.5
Not applicable (HER2–)	155	61.0
MP system		
1	19	7.5
2	58	22.8
3	52	20.5
4	45	17.7
5	80	31.5
RCB system		
0(pCR)	59	23.2
I	33	13.0
II	91	35.8
III	71	28.0

*Abbreviations*: *RFS* Relapse-free survival, *HER2* Human epidermal growth factor receptor 2, *HR* Hormone receptor, *TNBC* Triple negative breast cancer, *NAC* Neoadjuvant systemic therapy, *MP* Miller-Payne, *RCB* Residual cancer burden, *pCR* Pathologic complete response

In 254 patients, 59 cases showed disease progression, with 26 cases (44.1%) being ≤ 50 years old and 33 cases (55.9%) being > 50 years old.Menopausal status: 32 cases (54.2%) were premenopausal, and 27 cases (45.8%) were postmenopausal.Clinical stage: 19 cases (32.2%) were stage II, and 40 cases (67.8%) were stage III.Estrogen receptor: 23 cases (39%) were negative, and 36 cases (61%) were positive.Progesterone receptor: 31 cases (52.5%) were negative, and 28 cases (47.5%) were positive.HER2 status: 37 cases (62.7%) were negative, and 22 cases (37.3%) were positive.Ki-67 proliferation index: 16 cases (27.1%) had an index ≤ 20, and 43 cases (72.9%) had an index > 20.Receptor status: 23 cases (39%) were HR + /HER2-, 13 cases (22%) were HR + /HER2 + , 9 cases (15.3%) were HR-/HER2 + , and 14 cases (23.7%) were TNBC.Type of breast surgery: 55 cases (93.2%) underwent mastectomy, and 4 cases (6.8%) underwent BCS. Pathologic T stage after NAC): 23 cases (39%) were ypT0, 18 cases (30.5%) were ypT1, 13 cases (22%) were ypT2, 2 cases (3.4%) were ypT3, and 3 cases (5.1%) were ypT4.Pathologic N stage after NAC: 14 cases (23.7%) were ypN0, 16 cases (27.1%) were ypN1, 19 cases (32.3%) were ypN2, and 10 cases (17%) were ypN3.Lymphatic vessel invasion (LVI): 42 cases (71.2%) were negative, and 17 cases (28.8%) were positive. NAC regimen: 4 cases (6.8%) received anthracycline, 6 cases (10.2%) received taxane, and 49 cases (83%) received anthracycline + taxane.Neoadjuvant anti-HER2 therapy regimen: 12 cases (20.3%) rejected treatment, 8 cases (13.6%) received a single targeted drug, 2 cases (3.4%) received double targeted drugs, and 37 cases (62.7%) were not applicable (HER2-).Types of progression: 7 cases (11.9%) had local recurrence, and 52 cases (88.1%) had distant metastasis.MP system: 9 cases (15.3%) were grade 1, 17 cases (28.8%) were grade 2, 15 cases (25.4%%) were grade 3, 10 cases (16.9%) were grade 4, and 8 cases (13.6%) were grade 5.RCB system: 6 cases (10.2%) were RCB 0 (pCR), 2 cases (3.4%) were RCBI, 22 cases (37.3%) were RCBII, and 29 cases (49.1%) were RCBIII( as shown in sTable 1).

### Correlation of factors with RFS

The statistical results in Table 2 show that the age at the first diagnosis(*P* = 0.005), clinical stage (*P* = 0.005), Pathologic T stage after NAC (*P* < 0.001),Pathologic N stage after NAC (*P* < 0.001), LVI (*P* < 0.001),and RCB (*P* < 0.001) were significant characteristics for RFS. Then, these 6 significant factors were included in the multivariate Cox regression model, and age (HR of > 50 years vs. ≤ 50 years = 1.891; *P* = 0.022), Pathologic T stage after NAC (*P* = 0.023), and RCB score (*P* = 0.003) were significant.

**Table 2:** Analysis of correlation factors with RFS

Factors	Univariate analysis			Multivariate analysis		
All patients(*n* = 254)	HR	95% CI	*P* Value	HR	95% CI	*P* Value
Age at diagnosis						
≤ 50	1			1		
> 50	2.097	1.254–3.507	0.005	1.891	1.098–3.257	0.022
Clinical stage						
II	1			1		
III	2.171	1.256–3.751	0.005	1.597	0.875–2.913	0.127
Pathologic T stage after NAC			< 0.001			0.023
ypT0	1			1		
ypT1	1.157	0.623–2.148	0.645	0.592	0.288–1.216	0.154
ypT2	1.432	0.724–2.832	0.302	0.542	0.240–1.221	0.139
ypT3	10.484	2.445–44.957	0.002	2.023	0.389–10.512	0.402
ypT4	10.677	3.161–36.058	< 0.001	3.837	0.996–14.777	0.051
Pathologic N stage after NAC			< 0.001			0.167
ypN0	1			1		
ypN1	2.084	1.015–4.278	0.045	1.354	0.565–3.247	0.497
ypN2	4.431	2.218–8.850	< 0.001	1.933	0.811–4.604	0.137
ypN3	7.267	3.213–16.439	< 0.001	2.895	1.064–7.873	0.037
LVI						
Negative	1			1		
Positive	2.755	1.566–4.847	< 0.001	1.384	0.748–2.562	0.300
RCB system			< 0.001			0.003
0	1			1		
I	0.581	0.117–2.880	0.506	0.712	0.141–3.591	0.680
II	2.601	1.054–6.418	0.038	3.270	1.228–8.707	0.018
III	4.946	2.051–11.926	< 0.001	5.108	1.838–14.199	0.002

*Abbreviations*: *LVI* Lymphatic vessel invasion

**Fig. 2:**
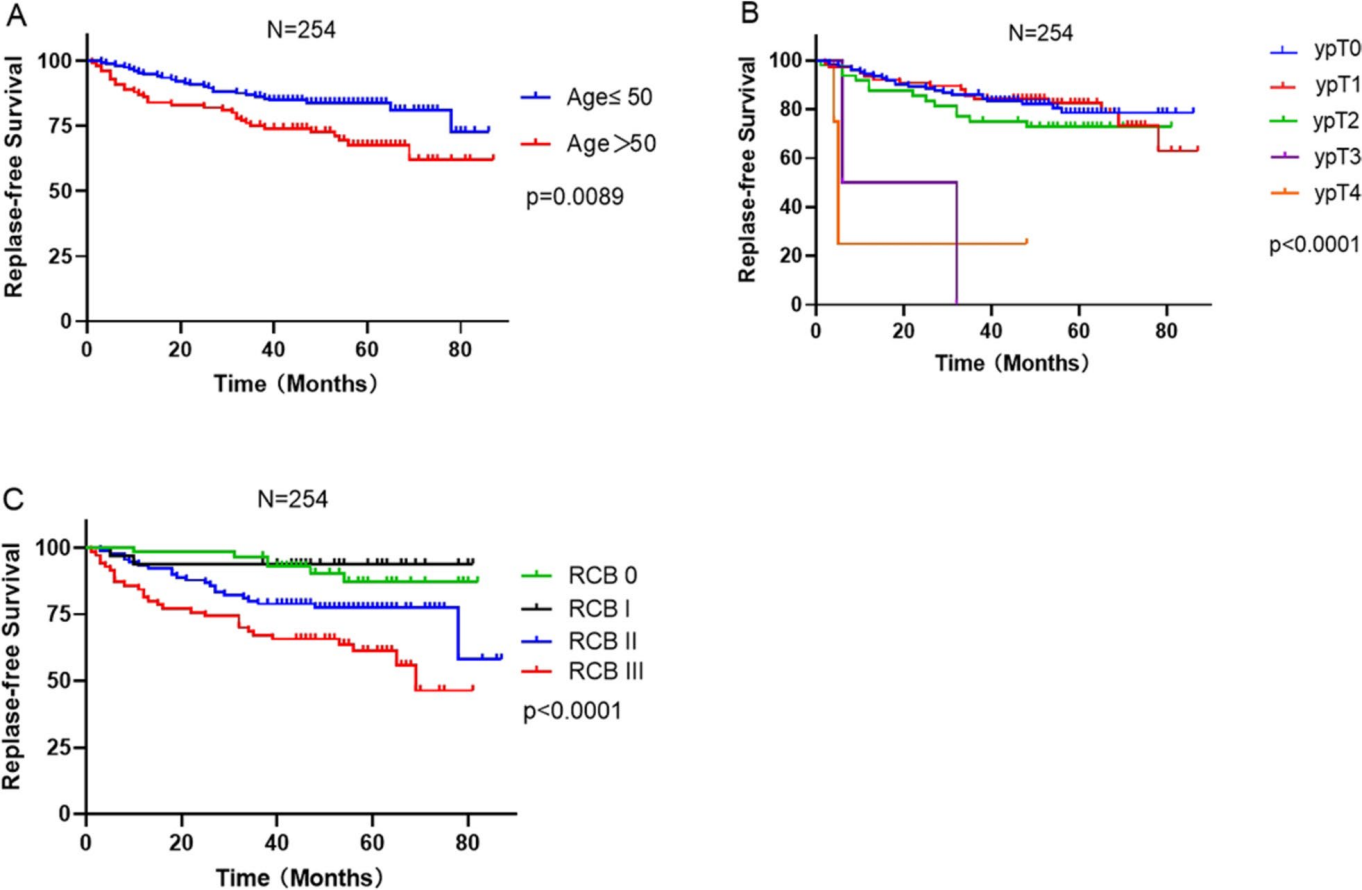
Kaplan–Meier estimates of RFS with different influencing factors. (**A**) Age at diagnosis, (**B**) Pathologic T stage after NAC, (**C**) RCB classes

Kaplan–Meier (K-M) survival curves for RFS based on the age, pathologic T stage after NAC, and RCB score are displayed in Fig. 2. The analysis suggest that age > 50 years (Fig. 2A), high pathologic T stage after NAC (Fig. 2B) and a high RCB score(Fig. 2C) were significantly correlated with a shorter time to relapse. Notably, in the risk assessment, there was apparently no difference in RCB I compared with RCB 0 (*P* = 0.680, Table 2).

### Correlation of factors with pCR

Among 254 patients, 59 (23.2%) achieved pCR (breast and axilla negative). To assess the association between pCR and clinicopathologic factors, we conducted logistic regression analysis. In a multivariate model, significant predictors of pCR included the TNBC and HER2 + subtypes. In total, 86.6% of cases received an anthracycline/taxane-based regimen with no association found between pCR and the type of chemotherapy (*P* = 0.310, Table 3).

**Table 3:** Association of Factors with pCR

Correlation of factors with pCR	Univariate analysis			Multivariate analysis		
Factors	OR	95% CI	*P* Value	OR	95% CI	*P* Value
Age at diagnosis (> 50 years)						
≤50 years	1			1		
>50 years	1.127	0.624–2.034	0.692	1.007	0.540–1.878	0.982
Clinical stage						
II	1			1		
III	0.704	0.392–1.264	0.240	0.720	0.388–1.333	0.296
HR status						
HR + /HER2 –	1			1		
HR + /HER2 +	1.845	0.752–4.530	0.181	1.858	0.748–4.615	0.182
HER2 +	4.026	1.827–8.871	0.001	3.608	1.580–8.239	0.002
TNBC	3.019	1.317–6.922	0.009	2.890	1.253–6.665	0.013
Chemotherapy type						
other regimens	1			1		
Anthracycline+ taxane	0.498	0.230–1.080	0.077	0.646	0.277–1.504	0.310

### RCB reclassification based on different prognoses

**Fig. 3:**
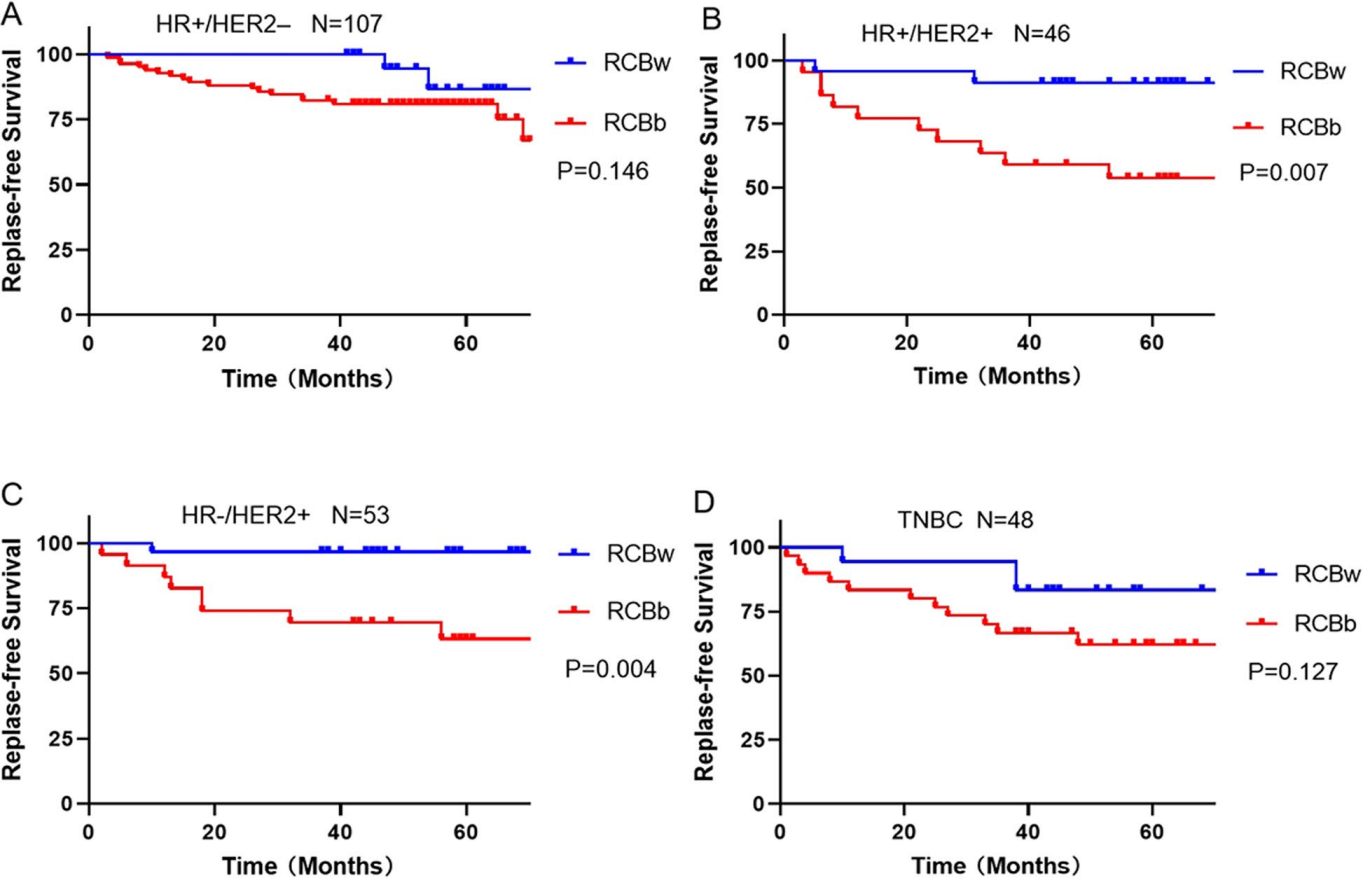
Replase-free survival by subtype based on reclassified RCB. (**A**) HR+/HER2-. (**B**) HR+/HER2+. (**C**) HR-/HER2+. (**D**) TNBC. RCBw: RCB0/I; RCBb: RCBII/III

According to the K-M curve of RCB for RFS, we classified RCB into two categories: RCB 0/RCB I with good prognosis was denoted as RCBw, and RCB II/RCB III with poor prognosis was denoted as RCBb. To further verify the predictive effect of RCB in each molecular type, we carried out K-M survival curve analysis. The results are shown in Fig. 3. In HR + /HER2+ and HR-/HER2 + types, the prognosis of RCBb was worse than that of RCBw(*p* = 0.007; *P* = 0.004, respectively). However, there were no statistical differences in the HR + /HER2- and TNBC subtypes with *P* = 0.146 and *P* = 0.127.

### ROC curves for RCB and Miller-Payne grade

**Fig. 4:**
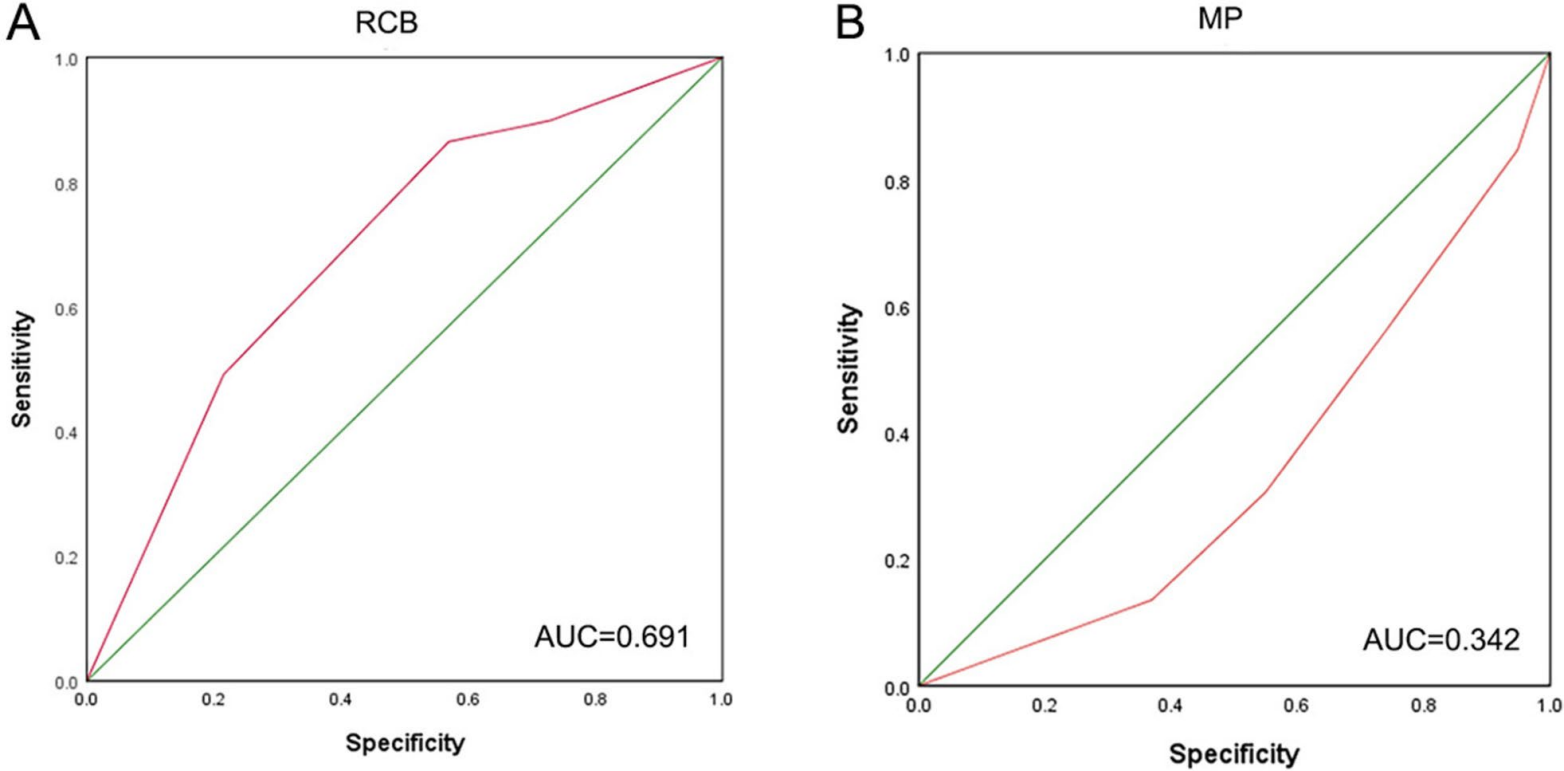
RCB and the Miller-Payne scoring systems serve as prognostic predictors for breast cancer following NAC. (A) ROC curve analysis of the RCB scoring system.(B) ROC curve analysis of the Miller-Payne scoring system

To verify the predictive effect of the RCB and Miller-Payne systems on RFS after NAC, we performed an ROC curve analysis, which show that the AUCs of the RCB and Miller-Payne systems were 0.691 and 0.342, respectively. This result indicates that both of them are effective evaluation systems for RFS, but the specificity and sensitivity of the RCB score to RFS were stronger than those of the Miller-Payne P grading system (Fig. 4).

## Discussion

Breast cancer includes multiple molecular subtypes and is a highly heterogeneous solid tumor [21]. NAC has been proven to increase the radical resection rate and breast preservation rate. The treatment efficacy varies from person to person, and the clinical response is a method of early evaluation [22, 23].In this paper, we emphatically analyzed the viability of the RCB model and its influence on the prognosis after NAC. Our cohort was a collection of high-risk cases (Table 1: TNBC 18.9% and HER2-positive 20.9%), and the distribution of these subtypes was similar to that in the RCB validation groups performed by Symmans et al. [14, 16]. Although the research has some shortcomings, we found that the survival prediction of RCB was similar to that in previous studies.

The Miller-Payne grading system is an accepted model that compares preoperative and postoperative tumor tissues, and is extensively used in neoadjuvant efficacy evaluation in domestic hospitals [24]. According to the percentage of cell density reduction in primary tumor foci, the system categorizes NAC efficacy from class 1 to class 5 [25]. Although it concisely and visually depicts the critical parameters associated with breast carcinomas and guides the selection of subsequent clinical treatment, it does not meticulously assess the postoperative pathology, particularly in patients with lymph node metastasis. And the MP system is not sufficiently comprehensive to measure the curative effect of tumor treatment due to the evaluation of only primary breast lesions. Moreover, following effective NAC for smaller tumors, the decrease in tumor cell density is more obvious than that in larger tumors, which indicates that the change in tumor cell density alone is not sufficiently comprehensive and objective to evaluate the therapeutic effect of tumor treatment [26]. In contrast, the RCB system has more meticulous requirements for specimen collection and microscopic evaluation after NAC. The RCB score contains information on the tumor foci and positive lymph nodes. The long and short diameters of the tumor foci, number of positive lymph nodes, proportion of the primary tumor beds that contain infiltrating cells and maximum diameter of the axillary lymph node metastasis are used to calculate the score after the NAC [27]. And this system has been gradually recognized in China over the years.

As an effective postoperative pathological response evaluation system, RCB has been validated in many countries and regions. A classic clinical test (protocol MDACC-LAB98–240), with the longest cohort follow-up time of 13 years, revealed no difference in survival between low RCB grades; in contrast, poor prognosis was mainly associated with the higher RCB class, which was assessed by Symmans et al. [16]. This conclusion was validated in another study by Müller, H. D et al.,who enrolled 184 cases [15]. In our retrospective study, we found that patients classified as RCB I (HR of RCB I vs. RCB 0 = 0.712, *p* = 0.680) could have a good prognosis and a low risk of recurrence. As expected, patients with a higher RCB class had worse survival outcomes, as confirmed by the Cox multivariate analysis, where RCB II (HR of RCB II vs. RCB 0 = 3.270, *p* = 0.018) and RCB III (HR of RCB III vs. RCB 0 = 5.108, *p* = 0.002) were significant factors. The results are consistent with those obtained by others. Ki-67 represents cell proliferation and is a recognized risk factor in breast cancer patients [28, 29]. Our study show that patients had a shorter RFS with high pathologic T stage after NAC, which suggest that lesions with high proliferative capacity may have worse outcomes. The results are also consistent with findings in other studies, Li-Yun Xie et al. revealed that a higher pre-neoadjuvant clinical T stage and N stage were independent predictors for an increased risk of tumor recurrence. Similar research results have also been found by Mariko Asaoka, supporting the conclusion of our study [30, 31].

Young age is a known risk factor for long-term survival in patients who undergo BCS and are not treated with NAC [32, 33]. This view was verified by a meta-analysis of large-scale prospective tests of BCS, which suggested that younger female patients had a higher 10-year locoregional recurrence rate(LRR) [34]. Nevertheless, once the patients were treated with NAC, we could not able to assess the role of age in predicting survival outcomes. A large and authoritative EORTC 10994/BIG 1–00 study showed that younger age was not a risk factor for local recurrence(LR) [35], and another study by Müller, H. D et al. from Europe did not separately analyze the age [15]. Another study included 263 cases, with a cutoff value of 50 years, and mainly analyzed the impact of younger age on LR after NAC. The results revealed that patients < 50 years could have higher pCR rates, and young age could have a better outcome after NAC [36]. Our study divided the cases by age into two sets, with 102 patients (40.2%) > 50 years, and we concluded that older age (> 50 years) would have a higher rate of relapse; however, we have no evidence to verify that younger age was highly predictive of pCR.

Our binary logistic regression analysis reveal that the phenotypic subtype was the unique associated factor in models that included age, stage, and chemotherapy regimens, and we found that patients with HER2-positive breast cancer, particularly TNBC, had higher pCR rates than HR-positive/HER2-negative patients. Similar conclusions have been observed in other studies [37, 38]. Increased RFS with pCR occurred regardless of the clinicopathological characteristics, including HR-positive/HER2-negative patients [39]. Finally, ROC curves were used to evaluate the prognostic efficiency of the RCB and Miller-Payne scoring systems for RFS, including calculation of the AUC, which demonstrated the favorable diagnostic efficiencies of the RCB and Miller-Payne scoring systems, with AUCs of 0.691 and 0.342, respectively. Taken together, these data suggest that the two systems are promising predictors for breast cancer patients treated with NAC.

According to the "NCCN Guidelines Version 2023 Breast Cancer," for TNBC patients who do not achieve pCR, oral capecitabine for 1 year may be considered as a treatment option. Multiple studies on stage III disease have shown that postoperative radiation therapy can improve local control, even for patients who have achieved pCR with NAC [40]. Additionally, the use of preoperative systemic therapy can provide important prognostic information based on treatment response. Extra attention should be given to patients with RCB grade 3. Other subtypes do not require further adjuvant treatment, but we can enhance their follow-up process and reduce the time needed for reviews.

However, there are some limitations in this study. The follow-up period is relatively short, while the survival time of breast cancer patients is relatively long. Therefore, we were unable to obtain the overall survival time of patients to include it in our research.In the future, we will continue to expand the number of case samples and increase the follow-up time to obtain more convincing survival data. Second, our study may have introduced selection bias because the data came from only two hospitals. Last, due to the diversity of NAC regimens, 86.6% of cases had received the same kind of chemotherapy regimen.

Although the RCB system has more detailed requirements for evaluation, it can only be used to evaluate postoperative pathology. The cell density of thick-needle aspiration specimens before NAC and surgical specimens after NAC can’t be compared as in the Miller-Payne system and can’t reflect the contrast gap before and after NAC, so it also has some limitations. Kim JY,et al.combined RCB score with the Ki67 to form a "residual proliferative tumor load" (residual proliferative cancer burden, RPCB) system, and the RPCB score provided richer prognostic information and had a higher predictive efficiency [41]. Recent studies have combined tumor infiltrating lymphocytes (TILs) with the RCB score as an original evaluation system, particularly in TNBC, which also shows good prospects [42, 43].

Our team analyzed the differentially expressed genes of the resected tissue following NAC, and it is believed that promising biomarkers with prognostic value will be found soon. In addition, Shandong Cancer Hospital took the lead in performing internal breast lymph node biopsy in China [44–48]. We also envision combining the internal breast lymph node information with the RCB system to develop a new, more comprehensive and accurate postoperative pathological evaluation system.

## Conclusions

Overall, the evaluation of pathologic response is immensely valuable because it provides a reliable supplement to pretreatment clinical and pathologic information. The RCB scoring system serves as an outstanding instrument to help us identify patients who are candidates for post-neoadjuvant clinical studies. We accomplished our research objective by proving that the RCB system as a pathological evaluation system after NAC is also applicable in routine clinical settings.

### Supplementary Information

**Additional file 1:** **sTable 1.** Baseline characteristics of patients with disease progression.

## Data Availability

The datasets used and/or analysed during the current study are available from the corresponding author on reasonable request.

## Abbreviations

RCB: Residual cancer burden
NAC: Neoadjuvant chemotherapy
RFS: Replase-free survival
pCR: Pathologic complete response
TNBC: Triple-negative breast cancer
AUC: Area under curve
BCS: Breast-conserving surgery
CSCO: Chinese Society of Clinical Oncology
ER: Estrogen receptor
PR: Progesterone receptor
HER2: Human epidermal growth factor receptor
LVI: Lymphatic vessel invasion
DCIS: Ductal carcinoma in situ
ROC: Receiver operating characteristic
LRR: Locoregional recurrence rate
LR: Local recurrence
RPCB: Residual proliferative cancer burden
TILs: Tumor infiltrating lymphocytes
