# Supplementary material for: The residual cancer burden index as a valid prognostic indicator in breast cancer after neoadjuvant chemotherapy
Source: BMC Cancer. 2024 Jan 2;24:13. doi: 10.1186/s12885-023-11719-z (PMC10762907; doi:10.1186/s12885-023-11719-z)
Supplement: Supplementary file 1 — Additional file 1: sTable 1. Baseline characteristics of patients with disease progression. [file 12885_2023_11719_MOESM1_ESM.docx]

**sTable 1 Baseline characteristics of patients with** **disease progression**

| Patient Characteristics |  |  |
| --- | --- | --- |
| Factors | N | Percentage(%) |
| Overall | 59 | 100.0 |
| Age at diagnosis  ≤50  ＞50 | 26  33 | 44.1  55.9 |
| Menopausal status  premenopausal  postmenopausal | 32  27 | 54.2  45.8 |
| Clinical stage  II  III | 19  40 | 32.2  67.8 |
| Estrogen receptor  Negative  Positive | 23  36 | 39.0  61.0 |
| Progesterone receptor  Negative  Positive | 31  28 | 52.5  47.5 |
| HER2 status  Negative  Positive | 37  22 | 62.7  37.3 |
| Ki-67 proliferation index  ≤20  ＞20 | 16  43 | 27.1  72.9 |
| Receptor status  HR+/HER2–  HR+/HER2+  HR-/HER2+  TNBC | 23  13  9  14 | 39.0  22.0  15.3  23.7 |
| Type of breast surgery  Mastectomy  Breast conservation | 55  4 | 93.2  6.8 |
| Pathologic T stage after NAC  ypT0  ypT1  ypT2  ypT3  ypT4 | 23  18  13  2  3 | 39.0  30.5  22.0  3.4  5.1 |
| Pathologic N stage after NAC  ypN0  ypN1  ypN2  ypN3 | 14  16  19  10 | 23.7  27.1  32.2  17.0 |
| Lymphatic vessel invasion(LVI)  Negative  Positive | 42  17 | 71.2  28.8 |
| Neoadjuvant chemotherapy regimen  Anthracycline  Taxane  Anthracycline+Taxane | 4  6  49 | 6.8  10.2  83.0 |
| Neoadjuvant anti-HER2 therapy regimen  reject  Single targeted drug  Double targeted drugs  Not applicable (HER2–) | 12  8  2  37 | 20.3  13.6  3.4  62.7 |
| Types of progression  Local recurrence  Distant metastasis | 7  52 | 11.9  88.1 |
| MP system  1  2  3  4  5 | 9  17  15  10  8 | 15.3  28.8  25.4  16.9  13.6 |
| RCB system  0(pCR)  I  II  III | 6  2  22  29 | 10.2  3.4  37.3  49.1 |
